# GST-4-Dependent Suppression of Neurodegeneration in *C. elegans* Models of Parkinson’s and Machado-Joseph Disease by Rapeseed Pomace Extract Supplementation

**DOI:** 10.3389/fnins.2019.01091

**Published:** 2019-10-17

**Authors:** Franziska Pohl, Andreia Teixeira-Castro, Marta Daniela Costa, Victoria Lindsay, Juliana Fiúza-Fernandes, Marie Goua, Giovanna Bermano, Wendy Russell, Patrícia Maciel, Paul Kong Thoo Lin

**Affiliations:** ^1^School of Pharmacy and Life Sciences, Robert Gordon University, Aberdeen, United Kingdom; ^2^Life and Health Sciences Research Institute (ICVS), School of Medicine, University of Minho, Braga, Portugal; ^3^ICVS/3B’s – PT Government Associate Laboratory, Braga, Portugal; ^4^Rowett Institute of Nutrition and Health, University of Aberdeen, Aberdeen, United Kingdom

**Keywords:** rapeseed (canola) pomace, *C. elegans*, Parkinson’s disease, Machado-Joseph disease, Spinocerebellar ataxia 3, antioxidant, gst-4, sod-3

## Abstract

Genetic mutations and aging-associated oxidative damage underlie the onset and progression of neurodegenerative diseases, like Parkinson’s disease (PD) and Machado-Joseph disease (MJD). Natural products derived from plants have been regarded as important sources of novel bioactive compounds to counteract neurodegeneration. Here, we tested the neuroprotective effect of an ethanolic extract of rapeseed pomace (RSP), a rapeseed (canola) oil production by-product, in *C. elegans* models of MJD and PD. The extract, containing sinapine and other phenolics, restored motor function of mutant ataxin-3 (ATXN3) animals (MJD) and prevented degeneration of dopaminergic neurons in one toxin-induced and two genetic models of PD. Whole-organism sensors of antioxidant and xenobiotic response activation revealed the induction of phase II detoxification enzymes, including glutathione S- transferase (GST-4) upon RSP extract supplementation. Furthermore *in vivo* pharmacogenetic studies confirmed *gst-4* is required for the therapeutic effect of RSP extract in the two disease models. The results suggest that GST-4-mediated antioxidant pathways may constitute promising therapeutic co-targets for neurodegenerative diseases and confirm the utility of searching for bioactive compounds in novel sources, including food and agricultural waste/by-products, such as RSP.

## Introduction

The number of people affected by neurodegenerative diseases has been increasing significantly over the last few decades, due to the ever-growing world population and an increase in life expectancy. Age is a major risk factor for neurodegeneration, hence the number of patients will further increase and become a major health issue worldwide. This is further aggravated by the lack of effective prevention and/or disease-modifying treatment strategies for these disorders. Drug discovery efforts are thus a priority (Joyner and Cichewicz, 2011; Ebrahimi and Schluesener, 2012).

Many neurodegenerative diseases, such as Alzheimer’s, Parkinson’s, and Huntington’s as well as other polyglutamine diseases, including Machado-Joseph disease (MJD), or Spinocerebellar ataxia type 3 (SCA3) have in common their association with aging, protein aggregation and oxidative stress (Gandhi and Abramov, 2012; Kim et al., 2015). In both familiar and sporadic forms of PD, mitochondrial dysfunction, neuroinflammation and environmental factors contribute to the susceptibility of dopaminergic neurons. When the production of Reactive Oxidative Species (ROS) surpasses the cellular antioxidant activity, the continuation of an oxidation state triggers cellular damage and causes neuronal loss, a process that is generally linked to normal aging but accelerated in disease states (Blesa et al., 2015). The involvement of oxidative stress in MJD is less well documented, however, it is thought that ATXN3 plays an important role in regulating the FOXO4-dependent antioxidant stress response *via* the manganese superoxide dismutase (SOD2), which is disrupted upon expression of the mutant protein. This suggests that a decreased antioxidative capacity and an increased susceptibility toward oxidative stress contribute to neuronal cell death in MJD (Araujo et al., 2011). In fact, MJD patients show decreased antioxidant defense capacity, as a result of increased ROS generation and decreased levels of glutathione peroxidase (de Assis et al., 2017), as well as reduced thiol levels (glutathione and thioredoxins) and increased DNA damage (Pacheco et al., 2013). This suggests that approaches to improve the cellular antioxidant capacity could lead to effective therapeutics.

Previous research on plant extracts and isolated compounds from plants (natural products) had shown promising results in both *in vitro* and *in vivo* models of normal aging (Ryu et al., 2016) as well as in models of neurodegenerative diseases (Joyner and Cichewicz, 2011; Ebrahimi and Schluesener, 2012; Pohl and Kong Thoo Lin, 2018). HPLC-MS/MS analysis of RSP extracts revealed the presence of different secondary metabolites, such as phenolic acids, benzaldehydes, amines, indoles, flavonoids and coumarins, sinapine being the most abundant secondary metabolite (Pohl et al., 2018; Yates et al., 2019). Sinapine is a known acetylcholinesterase inhibitor due to its structural similarity to acetylcholine (Ferreres et al., 2009) and has neuroprotective effects and lifespan increasing properties in several model systems (Li and Gu, 1999; Yang and He, 2008; Fu et al., 2016). We have previously shown the antioxidant and radical scavenging activity of the ethanolic rapeseed pomace (RSP) extract *in vitro*. In addition to the *in vitro* antioxidant activity, both the RSP extract and sinapine exhibited acetylcholinesterase inhibition activity and protected plasmid DNA from oxidative stress induced DNA damage *in vitro* (Pohl et al., 2018; Yates et al., 2019).

Our previous results suggested the direct antioxidant activity of the RSP extract. Direct antioxidants are redox active, short-lived and are consumed or chemically modified during the process of their antioxidant activity. They need to be regenerated or replenished for continuous activity. However, at certain concentrations they can also show pro-oxidant activity (Dinkova-Kostova and Talalay, 2008). In comparison, indirect antioxidants can activate antioxidant pathways, e.g., Kelch Like ECH Associated Protein 1/Nuclear factor (erythroid-derived 2)-like 2/antioxidant response elements (Keap1/Nrf2/ARE) in humans. This causes transcriptional induction of a variety of cytoprotective proteins, also known as phase II detoxification enzymes. For the activation of these pathways, the indirect antioxidants are not sacrificed and hence have a longer half-life (Dinkova-Kostova and Talalay, 2008; Christensen and Christensen, 2014). Different indirect antioxidants have been found in plants, such as curcumin in turmeric, carnosol and carnosic acid in the herb rosemary (Martin et al., 2004; Takahashi et al., 2009) and sulforaphane which is found in plants of the *Brassicaceae* family such as broccoli, cauliflower and cabbage (Dinkova-Kostova and Talalay, 2008; Visalli et al., 2017). While inducing cytoprotective proteins, some indirect antioxidants also act as direct antioxidants. By doing so they fulfill both roles, they can decrease ROS immediately and induce responses, which might have cytoprotective effects over a longer period of time (Dinkova-Kostova and Talalay, 2008).

In this work, to determine the *in vivo* antioxidant activity of RSP extract and its potential for the prevention of neurodegenerative diseases, the model organism *C. elegans* was used. Due to its numerous advantages (easy of culture in large numbers, a short life span and a well characterized nervous system, as well as its amenability to genetic manipulation and its transparency, even in adult stages), it is commonly used in neuroscience-related studies (Alexander et al., 2014; Chen et al., 2015; Ma et al., 2018). We focused on two neurodegenerative disease – PD and MJD – that have been associated with intracellular protein aggregation and in which oxidative stress has proven to be relevant. *C. elegans* models for both disorders have previously been created and employed for the search of potential treatments (Teixeira-Castro et al., 2011, 2015; Li et al., 2016; Manalo and Medina, 2018) and were used here to test the therapeutic potential of RSP extract, as a first *in vivo* evidence.

## Materials and Methods

### Strains and General Maintenance

A list of strains together with their abbreviations, genotype and source is given in Supplementary Table S1. All strains were cultured and observed using standard methods (Brenner, 1974) unless otherwise stated. *C. elegans* grew on Nematode Growth Medium (NGM) plates seeded with *Escherichia coli* OP50 strain at 20°C. All the strains were backcrossed to Bristol strain N2 six to eight times. The UA44 (Pdat-1:GFP; Pdat-1:α-syn) and UA57 (Pdat-1:GFP; Pdat-1:CAT-2) strains were generously provided by Guy Caldwell (University of Alabama). The MJD related strains (AT3q14, AT3q75, and AT3q130) were previously described (Teixeira-Castro et al., 2011) and double mutant strains [AT3q130;gst-4(ko) and α-syn;gst-4(ko)] were generated using common breeding techniques (Fay, 2013). The remaining strains were provided by the Caenorhabditis Genetics Center (CGC).

### Rapeseed Pomace Extracts and Sinapine

The RSP extract was prepared as previously described (Pohl et al., 2018; Yates et al., 2019). Although our previous studies have shown small batch-to-batch variability in chemical composition in extracts from different harvest years (Pohl et al., 2018), in this work several extractions were performed, and the obtained extracts collected, combined, homogenized, vacuum packed and stored at −80°C. Sinapine thiocyanate was obtained from ChemFaces, China (CFN90624) and was used without further purification.

### *C. elegans* Drug Toxicity Assay

The toxicity of distinct concentrations of RSP extract *in vivo* was determined in the wild-type N2 Bristol strain, using the food clearance assay (Voisine et al., 2007). The assay was performed as previously described (Voisine et al., 2007; Teixeira-Castro et al., 2015) in liquid culture in 96-well plate format using concentrations from 0.01–5.0 mg/mL of RSP extract and 0.001–1.0 mg/mL sinapine, using DMSO as the drug vehicle at a final concentration of 1%. Animals treated with 1% and 5% DMSO were used as a non-toxic (vehicle control) and as a toxic concentration control, respectively.

### Motor Performance and Mutant ATXN3 Aggregation in the *C. elegans* MJD Model Treated With RSP Extract

AT3q130 animals were treated with concentrations of RSP extract and sinapine ranging from 0.1 to 5.0 mg/mL and 0.001–1.0 mg/mL, respectively, in liquid culture in 96-well format as described for the toxicity assay (Voisine et al., 2007). The motility assay was performed as previously described (Teixeira-Castro et al., 2011), using *C. elegans* strains expressing WT (AT3q14) and mutant ATXN3 (AT3q130) proteins in their nervous system, as well as N2 as control. *In vivo c*onfocal dynamic imaging and quantification of mutant ATXN3 aggregates for the RSP extract treatment was conducted as previously described (Teixeira-Castro et al., 2011) using an Olympus FV1000 (Japan) confocal microscope, under a 60x oil (*NA* = 1.35) objective. Z-series images were acquired for vehicle (1% DMSO) and RSP extract-treated (4 mg/mL) animals, using a 515 nm laser excitation line for yellow fluorescent protein (YFP). Regarding quantification of the mutant ATXN3 aggregates, two parameters were measured: area of aggregates/total area and number of aggregates/total area in three repeated experiments. The RSP extract concentration chosen for the *in vivo* imaging (4 mg/mL) was based on the initial motility results.

### Quantitative Analysis of Dopaminergic Neuronal Degeneration in the 6-OHDA *C. elegans* PD Model Treated With RSP Extract

The experimental procedure for 6-hydroxydopamine (6-OHDA) exposure was adapted from Cao et al. (2005) with minor modifications, using the strain BZ555 that expresses green fluorescent protein (GFP) in all dopaminergic neurons. Briefly, an age synchronized egg population was obtained *via* bleaching (Stiernagle, 2006). Approximately 200 eggs were pipetted onto NGM plates, freshly seeded with freeze/thaw inactivated OP50 containing DMSO (1%, vehicle control) or RSP extract (4 mg/mL) (day 0). Approximately 48 h later (day 2), L2-L3 worms were washed off the plates and washed 2–3 times in distilled water (dH_2_O) containing 1% Luria broth (LB) until solution was clear (no remaining OP50). Worms were resuspended in approximately 500 μL of dH_2_O with 1% LB. In a 12-well plate, 250 μL of 6-OHDA (final concentrations 10 and 25 mM), 250 μL ascorbic acid (final concentrations 2 and 5 mM, respectively) and 500 μL of the worm solution were incubated for 1 h at ∼50 rpm on a Grant-Bio POS-300 orbital shaking platform. After this time, the worms were diluted and washed 2–3 times in dH_2_O with 1% LB. Thereafter, BZ555 worms were transferred back onto respective vehicle or RSP extract seeded plates. On day 5, approximately 72 h after 6-OHDA treatment, worms were prepared for confocal imaging, as described above. Dopaminergic (DAergic) neurons were counted in 10–12 animals and representative pictures of each condition were obtained. The experiment was repeated three times (total number of worms scored *n* ≥ 30).

### Quantitative Analysis of Dopaminergic Neuronal Loss in Alpha-Synuclein and CAT-2-Mediated PD Model Treated With RSP Extract

Age-synchronized populations of BZ555 (Pdat-1:GFP), UA44 (Pdat-1:GFP; Pdat-1:α-syn) and UA57 (Pdat-1:GFP; Pdat-1:CAT-2) strains were obtained *via* egg-laying. Briefly, gravid animals were picked onto plates seeded with freeze/thaw inactivated OP50 supplemented with vehicle (1% DMSO) and RSP extract (4 mg/mL) and left to lay eggs for 1–2 h before being removed from plates (day 0). After day 3, animals were transferred daily to fresh plates to avoid progeny. At day 7 and/or 10, worms (*N* = 10–12) were prepared for confocal microscopy as described above. Intact dopaminergic (DAergic) neurons were counted, and representative pictures of each condition were taken. The experiment was repeated independently three times (total number of worms scored *n* ≥ 30).

### Assessment of Antioxidant Response Induction Using Reporter Strains for *gst-4*, *sod-3* and *gcs-1* Genes

CL2166 [(pAF15)gst-4p:GFP:NLS], CF1553 [(pAD76) sod-3p:GFP + rol-6(su1006)], LD1171 [(gcs-1p:GFP + rol-6(su1006)] strains were grown in NGM plates seeded with freeze/thaw inactivated bacteria and RSP extract (4 mg/mL) until day 4 (∼96 h after hatching). Worms were prepared for fluorescence microscopy, by preparing 3% agar slides and adding a drop of sodium azide (2 mM) in addition to 10–12 worms per slide. Worms were oriented using an eye lash, excess azide was removed and the worms were covered with a cover slide and sealed with 3% agar. Brightfield (1.662 ms exposure time, ISO1600) and fluorescence (ISO1600, GFP filter) images of vehicle and RSP extract treated animals were acquired in an Olympus Microscope BX61 (10× objective) using the same respective settings. Fluorescence exposure time was set to a value where vehicle treated worms were barely visible and the same settings were used to analyze the RSP treated worms. Fluorescence intensity of each worm was measured using Fiji (ImageJ, 1.51n), divided by the total area of the respective animal and normalized to the mean of the vehicle treated worms. The experiment was repeated three times independently, *n* ≥ 10 being the number of animals analyzed per treatment in each experiment (per condition). For the purpose of showing the results, the same level of brightness and contrast was applied to vehicle- and RSP-treated animals, with no impact in the fluorescence quantification of the images.

### Statistical Analysis

All statistical analyses were performed using GraphPad Prism 7 (Version 7.01) or SPSS 25.0 (SPSS Inc.). Continuous variables were tested for normal distribution (Shapiro–Wilk or Kolmogorov–Smirnov normality test), for homogeneity of variance (Levene’s test) and outliers; and were then analyzed with one-way or two-way ANOVA, using Bonferroni’s, Dunnett’s or Tukey’s multiple comparison analysis for *post hoc* comparison. Non-continuous variables were analyzed through non-parametric Mann–Whitney or Kruskal–Wallis (with Bonferroni’s multiple comparison correction) tests, for two or more groups, respectively. For the comparison of effect sizes of RSP extract and sinapine treatments of experiments undertaken in independent trials, the Hedge’s test was done in R (version 3.6.1, package “effsize” version 0.7.6). A critical value for significance of *p* ≤ 0.05 was applied throughout the study. All experiments were run at least in triplicate (*n* ≥ 3) and data presented are showing mean ± standard deviation unless otherwise stated.

## Results

### RSP Extract Shows No Toxic Effect in *C. elegans*

**FIGURE 1:**
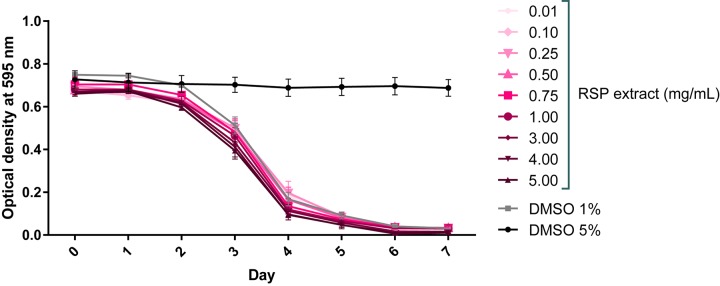
RSP extract (0.01–5.0 mg/mL) shows no toxic effect in *C. elegans*. Toxicity was assessed using the food clearance assay. The optical density of the OP50 suspension with RSP extract-treated animals (N2) at the concentrations depicted, was measured daily. The mean optical density (OD) was calculated for each day from triplicate samples and plotted over time. Control DMSO (1%) corresponds to drug vehicle and DMSO at 5% was used as positive (toxic compound) control, *n* = 5 (independent experiments).

RSP extract safety concentration range in *C. elegans* was determined by the food clearance assay (Voisine et al., 2007). A compound is considered safe to *C. elegans* if it causes no changes in animal growth, survival, and number of offspring, which can be measured indirectly by determining the rate of food consumption of the *E. coli* bacteria (OP50). The profile of the bacteria optical density curves obtained for all the tested concentrations of the RSP extract resembled that of vehicle treated animals (1% DMSO control, known to be safe) (Teixeira-Castro et al., 2015; Figure 1). The OP50 food source was cleared in an expected manner, starting on day 2–3 when worms were on the L4 molt. The further decrease in optical density on day 4 was associated with an increased number of worms in the wells, due to the appearance of progeny. There was no change in bacteria density upon 5% DMSO treatment, confirming its toxic effects. In summary, the RSP extract was considered safe to *C. elegans* at concentrations up to 5 mg/mL.

### Improvement of Motor Impairments of an MJD *C. elegans* Model With RSP Extract Treatment Independently of ATXN3 Aggregation Load

**FIGURE 2:**
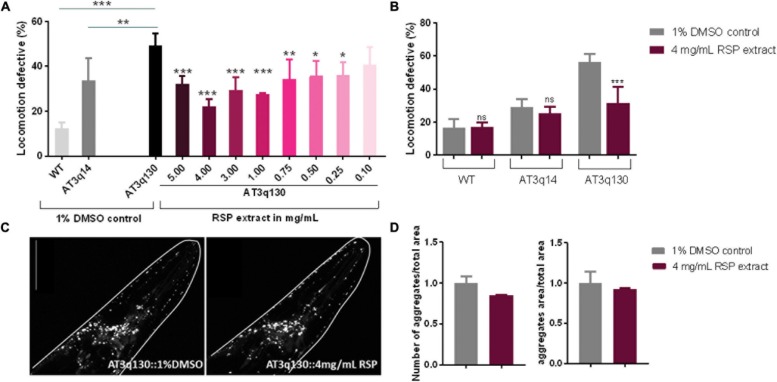
Improvement of motor impairments of the MJD *C. elegans* model with RSP extract treatment is independent of ATXN3 aggregation load. **(A)** Locomotion defective behavior of MJD (AT3q130) animals, comparison between treated (RSP extract 0.1–5.0 mg/mL), untreated animals (1% DMSO solvent control), wild type (N2) and AT3q14 controls (1% DMSO). Statistical significant difference was determined using One-way ANOVA and Dunnett’s multiple comparison analysis compared to AT3q130 control: ^∗∗∗^*p* ≤ 0.001, ^∗∗^*p* ≤ 0.01, ^∗^*p* ≤ 0.05; *n* = 5. **(B)** Comparison of locomotion defective behavior of N2, AT3q14, and AT3q130 untreated (1% DMSO vehicle control) and RSP extract treated (4 mg/mL) animals. Statistical significant difference determined using Two-way ANOVA and Tukey’s multiple comparisons test compared to untreated (1%DMSO) control: ^∗∗∗^*p* ≤ 0.001, ns-not significant, *n* = 6. **(C)** Confocal imaging of head region of AT3q130 strain treated with RSP (4 mg/mL, right) in comparison to vehicle control (1% DMSO, left). Confocal microscopy pictures are representative for the three independent experiments. **(D)** Aggregation load (number and area of aggregations) of AT3q130 animals upon treatment with RSP extract (4 mg/mL) compared to solvent control (1% DMSO). Values shown are the mean (normalized to vehicle treated control) of 10 or more animals per group; number of experiments *n* = 3, no significant difference, *p* > 0.05 (unpaired *t*-test). Data normalized to the 1% DMSO control. Scale bar 50 μm in all represented pictures.

To determine whether the RSP extract supplementation has therapeutic value for MJD, we used a *C. elegans* model of ATXN3 neurotoxicity (AT3q130) in which mutant ATXN3 proteins expressed in neurons, caused motility defects and aggregation (Teixeira-Castro et al., 2011). The results presented in Figure 2A demonstrated a concentration-dependent improvement in motility of the RSP-treated AT3q130 animals. Concentrations ranging from 1.00–5.00 mg/mL showed a very strong and significant improvement (*p* ≤ 0.001). However, lower concentrations of 0.75 mg/mL (*p* ≤ 0.01) and 0.50–0.25 mg/mL (*p* ≤ 0.05) also significantly improved the animals’ motor capacity. Only the lowest RSP extract concentration of 0.1 mg/mL showed no effect on the phenotype when administered to the animals. Treatment of AT3q130 animals with RSP extract at 4 mg/mL restored their motor function close to the level of WT ATXN3 expressing animals (AT3q14), hence this concentration was used in further experiments. To determine whether this motility improvement was disease specific, we also treated WT (N2) and AT3q14 animals with 4 mg/mL of RSP extract. The results confirmed a disease-specific effect since neither the N2 nor the AT3q14 motor performance changed upon treatment (Figure 2B).

As sinapine has been determined to be the most abundant compound present in the RSP extract (Yates et al., 2019), we next tested it for toxicity and disease modifying properties in AT3q130 animals. Sinapine showed no toxicity toward *C. elegans* up to 1 mg/mL (Supplementary Figure S1A) and had a beneficial effect in the disease model, although its effect in motility improvement was smaller (Hedge’s *g* = −3.967) than the RSP extract (Hedge’s *g* = −5.585) (Supplementary Figure S1B and Figure 2A), suggesting that additional components of the extract are contributing to its therapeutic effect. Hence, subsequent analyses were carried out using the whole RSP extract.

In MJD, the mutant ATXN3 (mATXN3) protein forms aggregates within affected regions of the patient’s brain. This is also observed in the *C. elegans* AT3q130 model (Teixeira-Castro et al., 2011), which shows mATXN3 aggregation in neuronal cells in the head region as well as in the dorsal and ventral nerve cords of the animals. Confocal imaging revealed no significant change in the number or area of mATXN3 protein aggregates in the head of treated animals compared to the untreated animals (Figures 2C,D), which demonstrates an uncoupling of RSP extract effect between these two main hallmarks of MJD, neuronal dysfunction and mutant protein aggregation.

### RSP Extract Treatment Protects From 6-OHDA-Induced Dopaminergic (DAergic) Neuronal Loss

**FIGURE 3:**
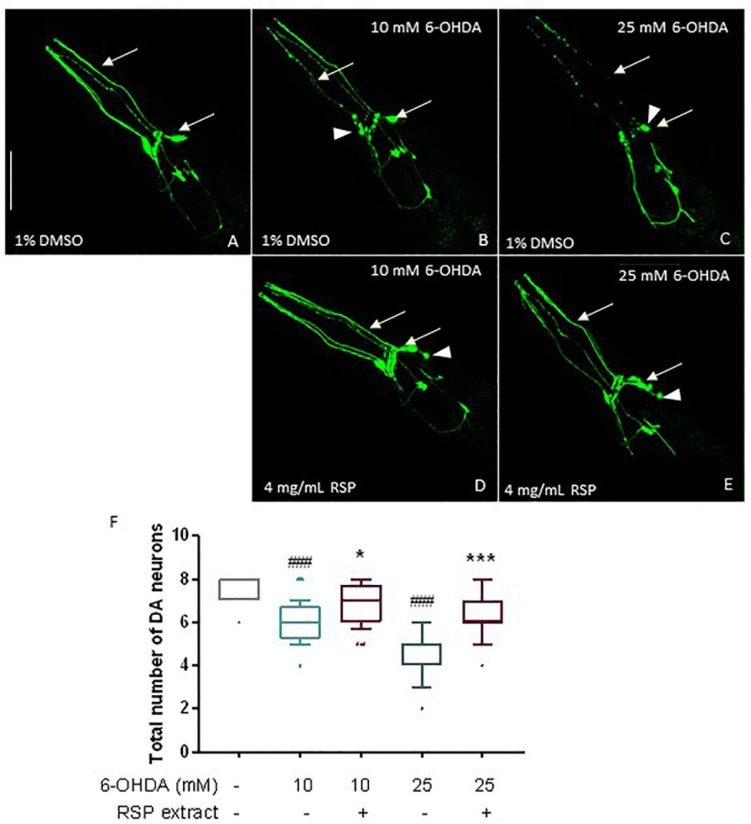
Protective effects of RSP extract supplementation (4 mg/mL) on DAergic neurodegeneration in *C. elegans*. *C. elegans* strain with GFP expression in all DAergic neurons (Pdat-1:GFP) grown in media supplemented with 1% DMSO (solvent control) and treated with **(A)**: 0 mM 6-OHDA and 0 mM AA, **(B)**: 10 mM 6-OHDA and 2 mM AA, **(C)**: 25 mM 6-OHDA and 5 mM AA. *C. elegans* strain expressing GFP proteins in all DAergic neurons (Pdat-1:GFP) grown in media supplemented with **(D,E)** 4 mg/mL RSP extract pre- and post- treated with **(D)**: 10 mM 6-OHDA and 2 mM AA, **(E)** 25 mM 6-OHDA and 5 mM AA. The fluorescence signals of DAergic neurons in the animals’ head (CEPs and ADEs) were photographed at 60× magnification using confocal fluorescence microscopy. Scale bar 50 μm in all represented pictures. **(F)** Total number of DAergic neurons were scored for each condition. Data are expressed as the median ± 10–90 percentile, with results obtained from three independent experiments (*n* = 3 with ≥ 10 animals per treatment). Significant differences between conditions were determined by non-parametric Kruskal–Wallis test and outlier analysis. ^#^*p* < 0.05, ^###^*p* < 0.001 compared with vehicle alone (no 6-OHDA), ns, ^∗^*p* < 0.05, ^∗∗^*p* < 0.01, and ^∗∗∗^*p* < 0.001 compared with respective 6-OHDA concentration control. 6-OHDA: 6-hydroxydopamine; AA: ascorbic acid; DAergic-dopaminergic neurons.

Given the promising results in the polyglutamine disease model described above, the effect of the RSP extract was next evaluated in a chemically induced model of PD. Transgenic animals expressing GFP proteins under the regulation of the dopamine transporter promoter (P*dat-1*:GFP) in all 8 hermaphrodite dopaminergic (DAergic) neurons (Figure 3A) were exposed to a mixture of 6-OHDA and ascorbic acid (as stabilizer) for 1 h. After 72 h, animals treated with 10 and 25 mM of 6-OHDA showed a clear loss of DAergic neurons (Figures 3B,C,F), compared to vehicle-treated control animals (Figures 3A,F) (*p* ≤ 0.001): *in vivo* dynamic imaging showed intact cell bodies and processes of the DAergic neurons of vehicle-treated GFP-expressing animals (Figure 3A), contrasting with a 6-OHDA concentration dependent increase in the blobbing of neuronal processes, and in the loss of cell bodies (Figures 3B,C). The four cephalic neurons (CEPs) were most susceptible to 6-OHDA treatment (Supplementary Figure S2).

When comparing the 1% DMSO with the 4 mg/mL RSP extract treated worms, there was a clear beneficial effect of the treatment (Figures 3B–E) for both 6-OHDA concentrations. Upon supplementation with RSP extract, more animals showed intact cell bodies and processes, although in some neurons the rounding of the cell body (Figure 3, arrowheads) was still visible upon treatment. This was confirmed when analyzing the total number of DAergic neurons (Figure 3F) as well as the CEP neurons alone (Supplementary Figure S1). These results suggest that supplementation with RSP extract protects against toxin-induced loss of DAergic neurons *in vivo*.

### RSP Extract Treatment Prevents DAergic Neuronal Loss in Genetic Models of PD

We then tested the effect of RSP extract treatment in genetically modified nematodes that were also previously described to mimic various aspects of PD neuropathology. Expression of human α-synuclein proteins (α-syn), in combination with GFP proteins, in the 8 *C. elegans* DAergic neurons induced neuronal death at day 7 and 10 of life (Cao et al., 2005; Cooper et al., 2006; Büttner et al., 2014). Overexpression of *cat-2*, a gene which encodes tyrosine hydroxylase, the limiting enzyme for dopamine synthesis in *C. elegans*, leads to increased intracellular levels of dopamine (Cao et al., 2005). As with α-synuclein, its overexpression is known to cause the degeneration of DAergic neurons in older animals. In *cat-2* overexpression animals, loss of DAergic neurons can also be detected by the co-expression of GFP in DAergic neurons (Cao et al., 2005; Masoudi et al., 2014).

**FIGURE 4:**
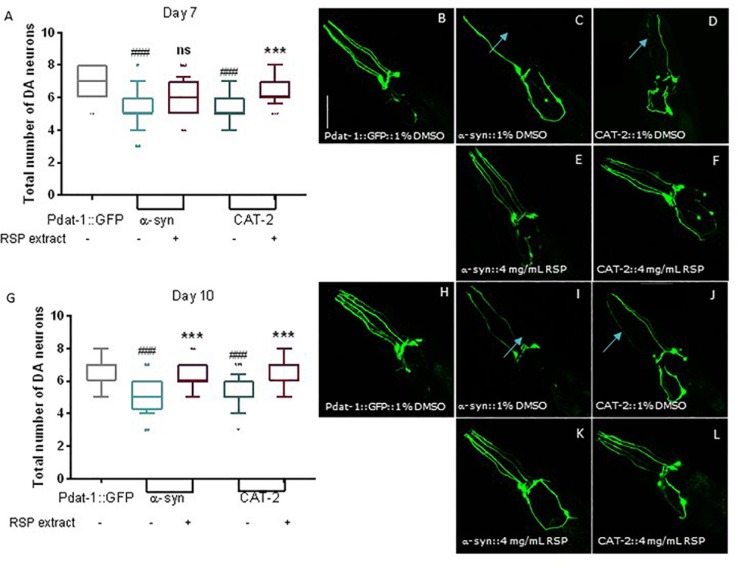
Supplementation with RSP extract suppressed loss of DAergic neurons mediated by alpha-synuclein and tyrosine hydroxylase overexpression in *C. elegans*. Confocal imaging and quantification of total number of DAergic neurons in Pdat-1:GFP animals (**A**, day 7 and **G**, day 10), in Pdat-1:GFP; Pdat-1:α-syn (**B**, day7 and **H**, day 10) and in Pdat-1:GFP; Pdat-1:cat-2 (**C**, day7 and **I**, day 10) vehicle treated (1% DMSO) animals, as well as in Pdat-1:GFP; Pdat-1:α-syn (**D**, day7 and **J**, day 10) and in Pdat-1:GFP; Pdat-1:cat-2 (**E**, day 7 and **K**, day 10) RSP extract (4 mg/mL) treated animals. Quantification of total number of DAergic neurons is shown in **F** (day 7) and **L** (day 10). Confocal microscopy pictures represent the head region of *C. elegans* and the pictures are representative of three independent experiments. Data is shown as the median ± 10–90 percentile with results obtained from three independent experiments (*n* = 3 with ≥ 10 animals per treatment). Significant differences between strains and treatment were determined by Kruskal–Wallis analysis, after outlier analysis: ns no significant difference. ^###^*p* < 0.001 compared with Pdat-1:GFP; ns ^∗∗∗^*p* < 0.001 compared with respective vehicle treated strain control. Scale bar 50 μm in all represented pictures.

Control animals expressing only GFP proteins in all DAergic neurons showed the presence of most neurons as well as the 4 dendrites associated with the 4 CEP neurons, heading to the front of the worm (Figures 4A,B; 7 days old animals and Figures 4G,H 10 days old animals). In contrast, animals overexpressing α-syn and CAT-2 proteins showed a significant loss of DAergic neurons and their processes on days 7 (Figures 4A,C,D) and 10 (Figures 4G,I,J). Animals treated with RSP extract for the duration of the experiment, showed an increase in the number of preserved DAergic cell bodies and dendrites, in particular for the CEPs, at day 7 (Figures 4A,E,F) and 10 (Figures 4G,K,L) indicating that the RSP extract treatment suppresses DAergic neuronal loss in the genetic PD model, as seen in the chemically induced model.

### Activation of Antioxidant Pathways by RSP Extract Treatment

**FIGURE 5:**
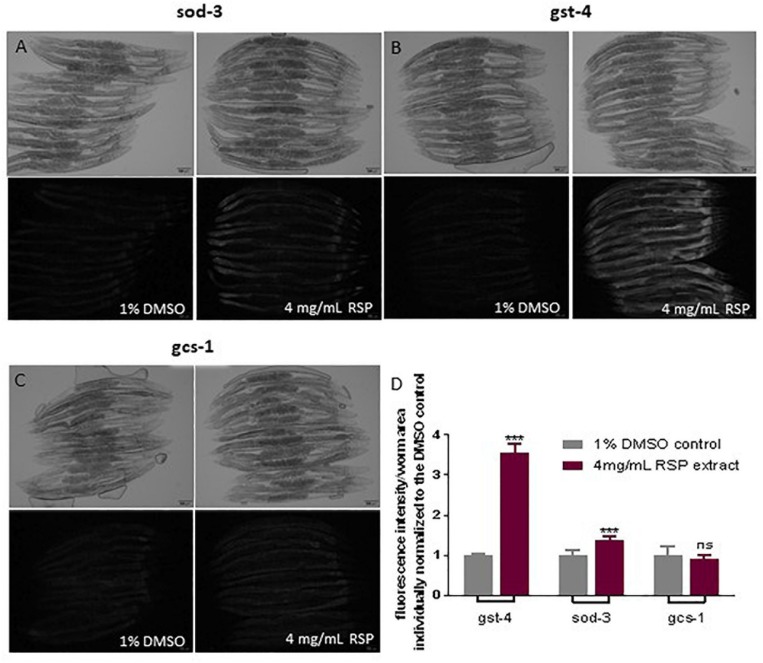
RSP extract supplementation activated transcriptional activity of *gst-4* and *sod-3*, but not of *gcs-1* gene promoters in *C. elegans* reporter strains. Brightfield and widefield fluorescence images of vehicle-(1% DMSO) and RSP-(4 mg/mL) treated Psod-3:GFP **(A)**, Pgst-4:GFP **(B)** and Pgcs-1:GFP **(C)** animals. Graphical results shown in **(D)** represents the GFP fluorescence intensity divided by the total area of each worm (*n* ≥ 9) normalized to the respective 1% DMSO control. Statistical comparison was done via two-way ANOVA and Bonferroni’s multiple comparisons test after Shapiro–Wilk normality test and outlier analysis, graph shows a representative example of three experimental replicates (*n* = 3). ns, no significant difference and ^∗∗∗^*p* < 0.001 compared with respective control. The pictures presented are from the same experimental day. Time of exposure is maintained constant in vehicle- and RSP-treated animals. Fluorescence intensity of each worm was measured using Fiji (ImageJ, 1.51n) and divided by the total area of the respective animal and normalized to the mean of the vehicle treated worms. Scale bar 100 μm in all represented pictures.

To test the hypothesis that the antioxidant properties of the RSP extract may contribute to the observed neuroprotective effect in *C. elegans* models of neurodegenerative diseases, we determined whether the RSP extract had the potential to activate antioxidant pathways, using *C. elegans* reporter strains for the *gst-4*, *gcs-1* and *sod-3* genes. GST-4 which encodes for the drug-metabolizing enzyme glutathione S-transferase 4 and *gcs-1*, the phase II detoxification enzyme gamma-glutamyl cysteine synthetase, are involved in glutathione metabolism and biosynthesis, respectively. Both genes are downstream targets of the transcription factor SKN-1 (Nrf-2 ortholog) (Hoeven et al., 2011). *sod-3* codes for the mitochondrial manganese superoxide dismutase, which converts the ROS superoxide into hydrogen peroxide. The transcriptional activity of these genes was measured indirectly by the expression of GFP. The results showed a modest but significant induction of the transcriptional activity of the *sod-3* gene promoter upon RSP treatment (Figures 5A,D). P*gst-4*:GFP animals show a three-fold induction of GFP fluorescence upon RSP extract treatment (Figures 5B,D), but no changes in the *gcs-1* promoter activity of Pgcs-1:GFP animals (Figures 5C,D) were observed in treated animals when compared with vehicle.

### Suppression of α-Synuclein- and Mutant ATXN3-Mediated Neurotoxicity by RSP Extract Treatment Is Dependent on GST-4

**FIGURE 6:**
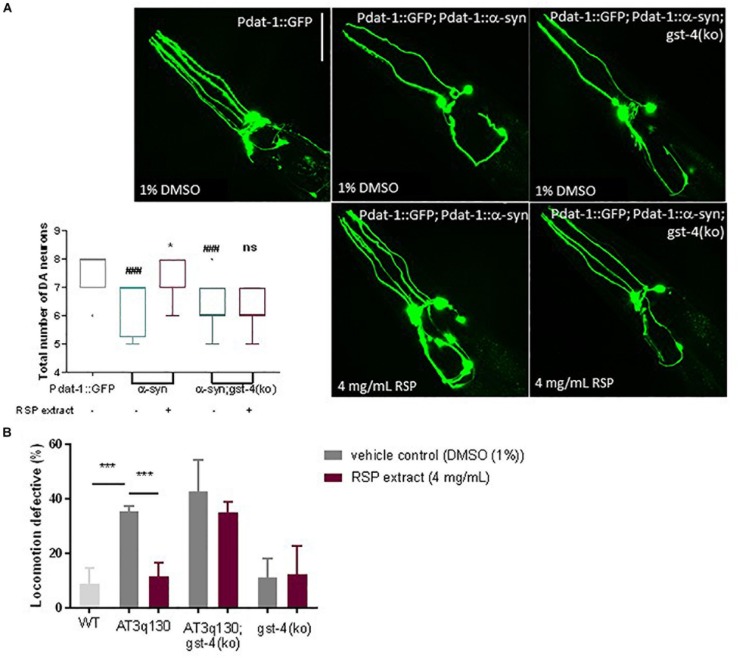
GST-4-dependent rescue of MJD and PD phenotypes by RSP extract supplementation. **(A)** DAergic neuron loss in Pdat-1:GFP, Pdat-1:α-syn and in Pdat-1:α-syn;gst-4(ko) animals comparing treated (RSP extract (4 mg/mL) and untreated [DMSO (1%)] conditions. Graph: 2 independent experiments (n) were conducted with 12 animals per group. Statistical significant difference was determined using the non-parametric Kruskal–Wallis test ^∗^*p* < 0.05 compared to untreated α-syn, ^###^*p* < 0.001 compared to Pdat-1::GFP, ns compared to untreated α-syn;gst-4(ko). Images: confocal imaging of DA neurons, scale bar 50 μm. **(B)** Motility assay comparison between WT, AT3q130, AT3q130; gst-4(ko) and gst-4(ko) strains. At least 200 animals were assayed per experiment, in five independent experiments (*n* = 5). Significant differences between strains and treatment were determined by one-way ANOVA and Bonferroni’s multiple comparison analysis, after Shapiro–Wilk normality test and outlier analysis: ^∗∗∗^*p* < 0.001 compared with respective control.

To determine whether the beneficial effects of RSP extract treatment in the *C. elegans* models of PD and MJD pathogenesis were mediated by GST-4, we assessed the effect of RSP extract supplementation in a *gst-4* deletion background [*gst-4(ko)*]. Ablation of *gst-4 per se* did not alter the α-syn-mediated neuronal loss (Figure 6A) or mutant ATXN3-mediated motor impairments (Figure 6B). However, RSP extract treatment failed to suppress α-syn-mediated loss of DAergic neurons in the absence of *gst-4* (Figure 6A), suggesting the importance of GST-4 activity in the extract’s mechanism of action.

A similar analysis made on AT3q130 animals in the background of *gst-4* ablation [AT3q130; *gst-4(ko)*] (Figure 6B) also indicated the role of GST-4 activation in the positive effect of the RSP extract in this disease model. AT3q130 animals showed significant motility improvements upon RSP extract treatment (4 mg/mL), whereas the AT3q130; *gst-4(ko)* double mutants showed no significant improvement in motor capacity compared to the vehicle control (1% DMSO). Taken together, these results support the idea that neuroprotection and suppression of proteotoxicity by RSP extract supplementation is dependent on the modulation of GST-4 activity in *C. elegans*.

## Discussion

In this study we describe the potential use of RSP extract to prevent neuronal dysfunction and neurodegeneration *in vivo* using transgenic *C. elegans* strains expressing mutant ATXN3, α-synuclein and tyrosine hydroxylase to mimic MJD and PD pathogenesis, respectively. In support of the findings, the loss of DAergic neurons was also rescued by RSP supplementation in a well-established toxin-induced model of PD. These models were previously employed to search potential treatments for these disorders, which were then proven effective in higher model organisms (Teixeira-Castro et al., 2011, 2015; Manalo and Medina, 2018; Yan et al., 2018).

Although MJD and PD are distinct clinical entities, they share common etiologies, such as the formation of protein aggregates (Dimitriadi and Hart, 2010) and some neuropathological mechanisms, including their link to mitochondrial dysfunction, oxidative stress and endoplasmic reticulum stress (Chakraborty et al., 2013; Chege and McColl, 2014). Increasing evidence suggested a possible interaction between proteins that have been classically associated to distinct neurodegenerative diseases (Wszolek et al., 2004; Lattante et al., 2014). Some of these diseases also share clinical and neuropathological features, an example being the slow movement or bradykinesia, that characterizes PD patients, and is also part of the clinical presentation of a subset of MJD patients. Some MJD patients also present parkinsonism with a positive response to levodopa (L-DOPA) (Bettencourt et al., 2011). Furthermore, α-synuclein aggregates were detected in the *substantia nigra* of MJD models (Alves et al., 2008; Noronha et al., 2017). This evidence suggests that common molecular and cellular pathways might underlie similarities between disease phenotypes, and that therapeutic compounds targeting those common aspects may be effective for both diseases.

Our study revealed a consistent neuroprotective effect of RSP extract in different models. In MJD, RSP extract supplementation restored motor function of the mutant ATXN3 animals, although not linked to a change in the ATXN3 aggregation load detected in live neurons. This dissociation between the impact on neuronal dysfunction and on ATXN3 aggregation was previously seen in a small molecule drug library screening based on the same animal model (Teixeira-Castro et al., 2015), and is consistent with many previous findings supporting such a dissociation for this and other polyglutamine diseases (Silva et al., 2011; Beam et al., 2012). Sinapine treatment contributes to the attenuation of the motor impairments of mutant ATXN3-expressing animals upon RSP supplementation, however, other components of the extract may also play a role, with potential synergic effects.

To our knowledge there have been no previous reports on the preventive effect of RSP extract in *C. elegans* models of neurodegenerative disease. However, other plant extracts and compounds isolated from plants have been investigated in *C. elegans* PD models. For example, the natural occurring polyamine spermidine rescued α-synuclein-induced loss of DAergic neurons (Büttner et al., 2014), through autophagy induction. A methanolic extract of red seaweed (*Chondrus crispus*) (Sangha et al., 2013) showed promising neuroprotective effects and decreased α-synuclein aggregation in muscle cells. These effects were associated with enhanced tolerance to oxidative stress but not to heat stress and with increased expression of *sod-3* and *skn-1*, the *C. elegans* ortholog of Nrf2 (Liu et al., 2015). In a MPP^+^-induced PD model, the extract of Korean mountain ash (*Sorbus alnifolia)* restored *C. elegans* viability and prevented neuronal loss (Cheon et al., 2016). That extract also showed neuroprotection upon CAT-2 overexpression, but with no impact on α-synuclein aggregation (Cheon et al., 2016).

Studies using natural occurring compounds for the potential treatment of polyglutamine diseases are less common. However, epigallocatechin-3-gallate (EGCG, a tea catechin) suppressed mutant ATXN3-mediated neurotoxicity with no impact on aggregation (Bonanomi et al., 2014). The natural product icariside II, a flavonol extracted from the *Epimedium* plant family, in contrast, ameliorated protein aggregation and proteotoxicity-mediated paralysis in a polyQ35 *C. elegans* model and increased the levels of *sod-3* and of *hsp-12.3*, a small heat shock protein that was described to prevent aggregate formation (Cai et al., 2011). Other plant secondary metabolites that induce the expression of GST-4 and of other enzymes involved in glutathione biosynthesis and metabolism, also reduce ROS accumulation, protect against oxidative stress and prolong organism lifespan (Hsu et al., 2012; Ogawa et al., 2016).

Overall, the impact of natural compounds on proteotoxicity has been primarily associated to their antioxidant properties since many studies reported activation of antioxidant genes/pathways. However, those studies fail to show a dependence of these proteins on their therapeutic role. Here, we report the induction of GST-4 expression by RSP extract and the lack of efficacy of RSP supplementation upon GST-4 ablation. Nevertheless, we do not exclude the role of additional cellular targets (e.g., SOD-3, other GSTs and other pathways), neither do we imply a direct dependency on the antioxidant activity of GST-4 *in vivo* on the therapeutic role of RSP extract. Endogenous modification of the catalytic domain/residue of GST-4 (e.g., by using CRISPR) would be necessary (Babbitt, 2000; Shi et al., 2015) for further elucidation. Other possible mechanisms of action remain to be tested for the RSP extract, including their role in the maintenance of the protein homeostasis capacity in neuronal cells (Balch et al., 2008) or in the increase of dopamine D2-like receptor (DOP2R) activation/availability (Manalo and Medina, 2018). To further understand the alteration the RSP extract is causing in transgenic *C. elegans* strains and to elucidate its role in MJD and PD, multi-omics combination including metabolomics, transcriptomics and proteomics would be a worthy strategy to adopt in the future.

In humans, glutathione transferases play crucial functions in the metabolism of xenobiotics and of endogenous compounds. In addition to their role in the detoxification of oxidative stress products, GST are also involved in the degradation of aromatic amino acids, synthesis of steroid hormones, synthesis and inactivation of eicosanoids and in the modulation of signaling pathways through their role in the metabolism of endogenous lipid mediators (reviewed in Hayes et al., 2005). The proposed human ortholog of GST-4 is hematopoietic prostaglandin D synthase (HPGDS) (Wormbase (Version WS272))^1^. This is a bifunctional enzyme of the sigma class of the glutathione-S-transferase family and catalyzes the conversion of PGH2 to PGD2 as well as the conjugation of glutathione with a number of electrophilic compounds (Kanaoka et al., 2000; Inoue et al., 2003; Flanagan and Smythe, 2011). GSTs have also been linked to a number of neurodegenerative diseases. In post-mortem brain samples of Alzheimer’s disease patients, GST activity and protein levels were significantly decreased, especially in the amygdala, the hippocampus, the inferior parietal lobule and the nucleus basalis of Meynert (Lovell et al., 1998). Furthermore, in a Drosophila PD model, genetic studies revealed that parkin mutants with additional (loss-of-function) mutations of GSTS1 genes [a sigma class GST (Singh et al., 2001)] caused a worse neurodegenerative phenotype than the parkin mutant itself (Whitworth et al., 2005). On the other hand, over-expression of GSTS1 in DAergic neurons suppressed neurodegeneration and hence, suggested a protective effect of these antioxidant enzymes in PD. Whether GST-4 in *C. elegans* has the same bifunctionality as seen for HPGDS is so far unclear and needs further research.

Despite the accumulation of evidence confirming the role of oxidants in several pathological conditions, most of the antioxidants developed for clinical use have been proven inconclusive or demonstrated limited success. This could be due to the fact that clinical trials include patients at broad stages of disease and therefore distinct severity of symptoms, as well as from different environmental and genetic backgrounds (Pohl and Kong Thoo Lin, 2018). The investigation of other sources of antioxidants, like RSP, and their application in earlier stages of disease progression and/or as a therapeutic adjuvant, as well as a closer monitoring of clinical trial design may improve therapeutic outcomes.

In conclusion, the RSP extract was shown to have positive effects *in vivo* in *C. elegans* models in a GST-4 dependent manner. This enzyme could constitute a viable therapeutic target for neurodegenerative diseases. For validation of this hypothesis prospective studies on the use of RSP extract or of the single compounds most prevalent in the extract, i.e., sinapine in vertebrate models of MJD and PD in comparison to other plant extracts should be conducted.

## Data Availability Statement

All datasets generated for this study are included in the manuscript/Supplementary Files.

## Author Contributions

FP, MG, GB, WR, PK, and PM contributed to the concept and discussion of using RSP extract as antioxidant therapy. FP, AT-C, MC, PK, and PM were instrumental to the conception and design of the *C. elegans* study. FP, AT-C, MC, VL, and JF-F performed the experiments. FP, AT-C, and VL performed the statistical analysis. FP, AT-C, PM, and PK helped in data interpretation. FP wrote the first draft of the manuscript. FP, AT-C, MC, and VL wrote sections of the manuscript. All authors contributed to manuscript revision, read, and approved the submitted version.

## Conflict of Interest

The authors declare that the research was conducted in the absence of any commercial or financial relationships that could be construed as a potential conflict of interest.

## Footnotes

1 https://www.wormbase.org/species/c_elegans/gene/WBGene00001752#0-9f-3 (accessed September 7, 2019).

## References

[B1] Alexander A. G. Marfil V. Li C. (2014). Use of *Caenorhabditis elegans* as a model to study Alzheimer’s disease and other neurodegenerative diseases. *Front. Genet.* 5:279 10.3389/fgene.2014.00279 PMC4155875 25250042

[B2] Alves S. Régulier E. Nascimento-Ferreira I. Hassig R. Dufour N. Koeppen A. (2008). Striatal and nigral pathology in a lentiviral rat model of Machado-Joseph disease. *Hum. Mol. Genet.* 17 2071–2083. 10.1093/hmg/ddn106 18385100

[B3] Araujo J. Breuer P. Dieringer S. Krauss S. Dorn S. Zimmermann K. (2011). FOXO4-dependent upregulation of superoxide dismutase-2 in response to oxidative stress is impaired in spinocerebellar ataxia type 3. *Hum. Mol. Genet.* 20 2928–2941. 10.1093/hmg/ddr197 21536589

[B4] Babbitt P. C. (2000). Reengineering the glutathione S-transferase scaffold: a rational design strategy pays off. *Proc. Natl. Acad. Sci. U.S.A.* 97 10298–10300. 10.1073/pnas.97.19.10298 10984526 PMC34038

[B5] Balch W. E. Morimoto R. I. Dillin A. Kelly J. W. (2008). Adapting proteostasis for disease intervention. *Science* 319 916–919. 10.1126/science.1141448 18276881

[B6] Beam M. Silva M. C. Morimoto R. I. (2012). Dynamic imaging by fluorescence correlation spectroscopy identifies diverse populations of polyglutamine oligomers formed in vivo. *J. Biol. Chem.* 287 26136–26145. 10.1074/jbc.M112.362764 22669943 PMC3406697

[B7] Bettencourt C. Santos C. Coutinho P. Rizzu P. Vasconcelos J. Kay T. (2011). Parkinsonian phenotype in Machado-Joseph disease (MJD/SCA3): a two-case report. *BMC Neurol.* 11:131. 10.1186/1471-2377-11-131 22023810 PMC3217914

[B8] Blesa J. Trigo-Damas I. Quiroga-Varela A. Jackson-Lewis V. R. (2015). Oxidative stress and Parkinson’s disease. *Front. Neuroanat.* 9:91 10.3389/FNANA.2015.00091 PMC4495335 26217195

[B9] Bonanomi M. Natalello A. Visentin C. Pastori V. Penco A. Cornelli G. (2014). Epigallocatechin-3-gallate and tetracycline differently affect ataxin-3 fibrillogenesis and reduce toxicity in spinocerebellar ataxia type 3 model. *Hum. Mol. Genet.* 23 6542–6552. 10.1093/hmg/ddu373 25030034

[B10] Brenner S. (1974). The genetics of *Caenorhabditis elegans*. *Genetics* 77 71–94. 10.1002/cbic.200300625 4366476 PMC1213120

[B11] Büttner S. Broeskamp F. Sommer C. Markaki M. Habernig L. Alavian-Ghavanini A. (2014). Spermidine protects against α-synuclein neurotoxicity. *Cell Cycle* 13 3903–3908. 10.4161/15384101.2014.973309 25483063 PMC4614020

[B12] Cai W.-J. Huang J.-H. Zhang S.-Q. Wu B. Kapahi P. Zhang X.-M. (2011). Icariin and its derivative icariside II extend healthspan via insulin/IGF-1 pathway in C. *elegans*. *PLoS One* 6:e28835. 10.1371/journal.pone.0028835 22216122 PMC3244416

[B13] Cao S. Gelwix C. C. Caldwell K. A. Caldwell G. A. (2005). Torsin-mediated protection from cellular stress in the dopaminergic neurons of *Caenorhabditis elegans*. *J. Neurosci.* 25 3801–3812. 10.1523/JNEUROSCI.5157-04.2005 15829632 PMC6724936

[B14] Chakraborty S. Bornhorst J. Nguyen T. T. Aschner M. (2013). Oxidative stress mechanisms underlying Parkinson’s disease-associated neurodegeneration in *C. elegans*. *Int. J. Mol. Sci.* 14 23103–23128. 10.3390/ijms141123103 24284401 PMC3856108

[B15] Chege P. M. McColl G. (2014). *Caenorhabditis elegans*: a model to investigate oxidative stress and metal dyshomeostasis in Parkinson’s disease. *Front. Aging Neurosci.* 6:89 10.3389/fnagi.2014.00089 PMC4032941 24904406

[B16] Chen X. Barclay J. W. Burgoyne R. D. Morgan A. (2015). Using *C. elegans* to discover therapeutic compounds for ageing-associated neurodegenerative diseases. *Chem. Cent. J.* 9 1–20. 10.1186/s13065-015-0143-y 26617668 PMC4661952

[B17] Cheon S.-M. Jang I. Lee M.-H. Kim D. K. Jeon H. Cha D. S. (2016). Sorbus alnifolia protects dopaminergic neurodegeneration in *Caenorhabditis elegans*. *Pharm. Biol.* 55 481–486. 10.1080/13880209.2016.1251468 27937005 PMC5490792

[B18] Christensen L. P. Christensen K. B. (2014). “The role of direct and indirect polyphenolic antioxidants in protection against oxidative stress,” in *Polyphenols in Human Health and Disease*, eds Watson R. R. Preedy V. R. Zibadi S., (Cambridge, MA: Academic Press), 289–309. 10.1016/B978-0-12-398456-2.00023-2

[B19] Cooper A. A. Gitler A. D. Cashikar A. Haynes C. M. Hill K. J. Bhullar B. (2006). Alpha-synuclein blocks ER-Golgi traffic and Rab1 rescues neuron loss in Parkinson’s models. *Science* 313 324–328. 10.1126/science.1129462 16794039 PMC1983366

[B20] de Assis A. M. Saute J. A. M. Longoni A. Haas C. B. Torrez V. R. Brochier A. W. (2017). Peripheral oxidative stress biomarkers in spinocerebellar ataxia type 3/Machado–Joseph disease. *Front. Neurol.* 8:485 10.3389/fneur.2017.00485 PMC5611390 28979235

[B21] Dimitriadi M. Hart A. C. (2010). Neurodegenerative disorders: insights from the nematode *Caenorhabditis elegans*. *Neurobiol. Dis.* 40 4–11. 10.1016/j.nbd.2010.05.012 20493260 PMC2926245

[B22] Dinkova-Kostova A. T. Talalay P. (2008). Direct and indirect antioxidant properties of inducers of cytoprotective proteins. *Mol. Nutr. Food Res.* 52 128–138. 10.1002/mnfr.200700195 18327872

[B23] Ebrahimi A. Schluesener H. (2012). Natural polyphenols against neurodegenerative disorders: potentials and pitfalls. *Ageing Res. Rev.* 11 329–345. 10.1016/j.arr.2012.01.006 22336470

[B24] Fay D. S. (2013). Classical genetic methods. *WormBook* 2013 1–58. 10.1895/wormbook.1.165.1 24395816 PMC4127492

[B25] Ferreres F. Fernandes F. Sousa C. ValentaÞo P. Pereira J. A. Andrade P. B. (2009). Metabolic and bioactivity insights into *Brassica oleracea* var. *acephala*. *J. Agric. Food Chem.* 57 8884–8892. 10.1021/jf902661g 19722523

[B26] Flanagan J. U. Smythe M. L. (2011). Sigma-class glutathione transferases. *Drug Metab. Rev.* 43 194–214. 10.3109/03602532.2011.560157 21425928

[B27] Fu R. Zhang Y. Guo Y. Peng T. Chen F. (2016). Hepatoprotection using *Brassica rapa* var. *rapa L*. seeds and its bioactive compound, sinapine thiocyanate, for *CCl*4-induced liver injury. *J. Funct. Foods* 22 73–81. 10.1016/J.JFF.2016.01.013

[B28] Gandhi S. Abramov A. Y. (2012). Mechanism of oxidative stress in neurodegeneration. *Oxid. Med. Cell. Longev.* 2012:428010. 10.1155/2012/428010 22685618 PMC3362933

[B29] Hayes J. D. Flanagan J. U. Jowsey I. R. (2005). GLUTATHIONE TRANSFERASES. *Annu. Rev. Pharmacol. Toxicol.* 45 51–88. 10.1146/annurev.pharmtox.45.120403.095857 15822171

[B30] Hoeven R. V. McCallum K. C. Cruz M. R. Garsin D. A. (2011). Ce-Duox1/BLI-3 generated reactive oxygen species trigger protective SKN-1 activity via p38 MAPK signaling during infection in *C. elegans*. *PLoS Pathog.* 7:e1002453. 10.1371/journal.ppat.1002453 22216003 PMC3245310

[B31] Hsu F.-L. Li W.-H. Yu C.-W. Hsieh Y.-C. Yang Y.-F. Liu J.-T. (2012). In vivo antioxidant activities of essential oils and their constituents from leaves of the Taiwanese *Cinnamomum osmophloeum*. *J. Agric. Food Chem.* 60 3092–3097. 10.1021/jf2045284 22380926

[B32] Inoue T. Irikura D. Okazaki N. Kinugasa S. Matsumura H. Uodome N. (2003). Mechanism of metal activation of human hematopoietic prostaglandin D synthase. *Nat. Struct. Mol. Biol.* 10 291–296. 10.1038/nsb907 12627223

[B33] Joyner P. M. Cichewicz R. H. (2011). Bringing natural products into the fold - exploring the therapeutic lead potential of secondary metabolites for the treatment of protein-misfolding-related neurodegenerative diseases. *Nat. Prod. Rep.* 28 26–47. 10.1039/c0np00017e 20927454

[B34] Kanaoka Y. Fujimori K. Kikuno R. Sakaguchi Y. Urade Y. Hayaishi O. (2000). Structure and chromosomal localization of human and mouse genes for hematopoietic prostaglandin D synthase. *Eur. J. Biochem.* 267 3315–3322. 10.1046/j.1432-1327.2000.01362.x 10824118

[B35] Kim G. H. Kim J. E. Rhie S. J. Yoon S. (2015). The role of oxidative stress in neurodegenerative diseases. *Exp. Neurobiol.* 24 325–340. 10.5607/en.2015.24.4.325 26713080 PMC4688332

[B36] Lattante S. Millecamps S. Stevanin G. Rivaud-Péchoux S. Moigneu C. Camuzat A. (2014). Contribution of ATXN2 intermediary polyQ expansions in a spectrum of neurodegenerative disorders. *Neurology* 83 990–995. 10.1212/WNL.0000000000000778 25098532 PMC4162303

[B37] Li H. Shi R. Ding F. Wang H. Han W. Ma F. (2016). Astragalus polysaccharide suppresses 6-hydroxydopamine-induced neurotoxicity in *Caenorhabditis elegans*. *Oxid. Med. Cell. Longev.* 2016:4856761. 10.1155/2016/4856761 27885333 PMC5112302

[B38] Li Q. Gu R. (1999). *The Effects of Sinapine from Cruciferous Plants on the Life-Span of Drosophila Melangaster.* Available at: http://en.cnki.com.cn/article_en/cjfdtotal-yyhs901.006.htm (accessed May 10, 2018).

[B39] Liu J. Banskota A. Critchley A. Hafting J. Prithiviraj B. (2015). Neuroprotective effects of the cultivated *Chondrus crispus* in a *C. elegans* model of Parkinson’s disease. *Mar. Drugs* 13 2250–2266. 10.3390/md13042250 25874922 PMC4413210

[B40] Lovell M. A. Xie C. Markesbery W. R. (1998). Decreased glutathione transferase activity in brain and ventricular fluid in Alzheimer’s disease. *Neurology* 51 1562–1566. 10.1212/wnl.51.6.1562 9855502

[B41] Ma L. Zhao Y. Chen Y. Cheng B. Peng A. Huang K. (2018). *Caenorhabditis elegans* as a model system for target identification and drug screening against neurodegenerative diseases. *Eur. J. Pharmacol.* 819 169–180. 10.1016/J.EJPHAR.2017.11.051 29208474

[B42] Manalo R. V. M. Medina P. M. B. (2018). Caffeine protects dopaminergic neurons from dopamine-induced neurodegeneration via synergistic adenosine-dopamine D2-like receptor interactions in transgenic *Caenorhabditis elegans*. *Front. Neurosci.* 12:137. 10.3389/fnins.2018.00137 29563862 PMC5845907

[B43] Martin D. Rojo A. I. Salinas M. Diaz R. Gallardo G. Alam J. (2004). Regulation of heme oxygenase-1 expression through the phosphatidylinositol 3-kinase/Akt pathway and the Nrf2 transcription factor in response to the antioxidant phytochemical carnosol. *J. Biol. Chem.* 279 8919–8929. 10.1074/jbc.M309660200 14688281

[B44] Masoudi N. Ibanez-Cruceyra P. Offenburger S.-L. Holmes A. Gartner A. (2014). Tetraspanin (TSP-17) protects dopaminergic neurons against 6-OHDA-induced neurodegeneration in *C. elegans*. *PLoS Genet.* 10:e1004767. 10.1371/journal.pgen.1004767 25474638 PMC4256090

[B45] Noronha C. Perfeito R. Laço M. Wüllner U. Rego A. C. (2017). Expanded and wild-type Ataxin-3 modify the redox status of SH-SY5Y cells overexpressing α-synuclein. *Neurochem. Res.* 42 1430–1437. 10.1007/s11064-017-2199-7 28236214

[B46] Ogawa T. Kodera Y. Hirata D. Blackwell T. K. Mizunuma M. (2016). Natural thioallyl compounds increase oxidative stress resistance and lifespan in *Caenorhabditis elegans* by modulating SKN-1/Nrf. *Sci. Rep.* 6:21611. 10.1038/srep21611 26899496 PMC4761942

[B47] Pacheco L. S. da Silveira A. F. Trott A. Houenou L. J. Algarve T. D. Belló C. (2013). Association between Machado–Joseph disease and oxidative stress biomarkers. *Mutat. Res. Toxicol. Environ. Mutagen.* 757 99–103. 10.1016/J.MRGENTOX.2013.06.023 23994570

[B48] Pohl F. Goua M. Bermano G. Russell W. R. Scobbie L. Maciel P. (2018). Revalorisation of rapeseed pomace extracts: an *in vitro* study into its anti-oxidant and DNA protective properties. *Food Chem.* 239 323–332. 10.1016/j.foodchem.2017.06.129 28873576

[B49] Pohl F. Kong Thoo Lin P. (2018). The potential use of plant natural products and plant extracts with antioxidant properties for the prevention/treatment of neurodegenerative diseases: *in vitro*, *in vivo* and clinical trials. *Molecules* 23:3283. 10.3390/molecules23123283 30544977 PMC6321248

[B50] Ryu D. Mouchiroud L. Andreux P. A. Katsyuba E. Moullan N. Nicolet-dit-Félix A. A. (2016). Urolithin A induces mitophagy and prolongs lifespan in C. elegans and increases muscle function in rodents. *Nat. Med.* 22 879–888. 10.1038/nm.4132 27400265

[B51] Sangha J. S. Fan D. Banskota A. H. Stefanova R. Khan W. Hafting J. (2013). Bioactive components of the edible strain of red alga, *Chondrus crispus*, enhance oxidative stress tolerance in *Caenorhabditis elegans*. *J. Funct. Foods* 5 1180–1190. 10.1016/J.JFF.2013.04.001

[B52] Shi J. Wang E. Milazzo J. P. Wang Z. Kinney J. B. Vakoc C. R. (2015). Discovery of cancer drug targets by CRISPR-Cas9 screening of protein domains. *Nat. Biotechnol.* 33 661–667. 10.1038/nbt.3235 25961408 PMC4529991

[B53] Silva M. C. Fox S. Beam M. Thakkar H. Amaral M. D. Morimoto R. I. (2011). A genetic screening strategy identifies novel regulators of the proteostasis network. *PLoS Genet.* 7:e1002438. 10.1371/journal.pgen.1002438 22242008 PMC3248563

[B54] Singh S. P. Coronella J. A. Beneš H. Cochrane B. J. Zimniak P. (2001). Catalytic function of *Drosophila melanogaster* glutathione S-transferase DmGSTS1-1 (GST-2) in conjugation of lipid peroxidation end products. *Eur. J. Biochem.* 268 2912–2923. 10.1046/j.1432-1327.2001.02179.x 11358508

[B55] Stiernagle T. (2006). Maintenance of C. elegans. *WormBook* 2006 1–11. 10.1895/wormbook.1.101.1 18050451 PMC4781397

[B56] Takahashi T. Tabuchi T. Tamaki Y. Kosaka K. Takikawa Y. Satoh T. (2009). Carnosic acid and carnosol inhibit adipocyte differentiation in mouse 3T3-L1 cells through induction of phase2 enzymes and activation of glutathione metabolism. *Biochem. Biophys. Res. Commun.* 382 549–554. 10.1016/j.bbrc.2009.03.059 19289108

[B57] Teixeira-Castro A. Ailion M. Jalles A. Brignull H. R. Vilaça J. L. Dias N. (2011). Neuron-specific proteotoxicity of mutant ataxin-3 in *C. elegans*: rescue by the DAF-16 and HSF-1 pathways. *Hum. Mol. Genet.* 20 2996–3009. 10.1093/hmg/ddr203 21546381 PMC3131043

[B58] Teixeira-Castro A. Jalles A. Esteves S. Kang S. da Silva Santos L. Silva-Fernandes A. (2015). Serotonergic signalling suppresses ataxin 3 aggregation and neurotoxicity in animal models of Machado-Joseph disease. *Brain* 138 3221–3237. 10.1093/brain/awv262 26373603 PMC4731417

[B59] Visalli G. Facciolà A. Bertuccio M. P. Picerno I. Di Pietro A. (2017). In vitro assessment of the indirect antioxidant activity of Sulforaphane in redox imbalance vanadium-induced. *Nat. Prod. Res.* 31 2612–2620. 10.1080/14786419.2017.1286485 28278681

[B60] Voisine C. Varma H. Walker N. Bates E. A. Stockwell B. R. Hart A. C. (2007). Identification of potential therapeutic drugs for huntington’s disease using Caenorhabditis elegans. *PLoS One* 2:e504. 10.1371/journal.pone.0000504 17551584 PMC1876812

[B61] Whitworth A. J. Theodore D. A. Greene J. C. Benes H. Wes P. D. Pallanck L. J. (2005). Increased glutathione S-transferase activity rescues dopaminergic neuron loss in a Drosophila model of Parkinson’s disease. *Proc. Natl. Acad. Sci. U.S.A.* 102 8024–8029. 10.1073/pnas.0501078102 15911761 PMC1142368

[B63] Wszolek Z. K. Pfeiffer R. F. Tsuboi Y. Uitti R. J. McComb R. D. Stoessl A. J. (2004). Autosomal dominant parkinsonism associated with variable synuclein and tau pathology. *Neurology* 62 1619–1622. 10.1212/01.wnl.0000125015.06989.db 15136696

[B64] Yan R. Zhang J. Park H.-J. Park E. S. Oh S. Zheng H. (2018). Synergistic neuroprotection by coffee components eicosanoyl-5-hydroxytryptamide and caffeine in models of Parkinson’s disease and DLB. *Proc. Natl. Acad. Sci. U.S.A.* 115 E12053–E12062. 10.1073/pnas.1813365115 30509990 PMC6304960

[B65] Yang C. Y. He L. (2008). Neuroprotective effects of sinapine on PC12 cells apoptosis induced by sodium dithionite. *Chin. J. Nat. Med.* 6 205–209. 10.1016/S1875-5364(09)60018-2

[B66] Yates K. Pohl F. Busch M. Mozer A. Watters L. Shiryaev A. (2019). Determination of sinapine in rapeseed pomace extract: its antioxidant and acetylcholinesterase inhibition properties. *Food Chem.* 276 768–775. 10.1016/J.FOODCHEM.2018.10.045 30409660

