# Supplementary material for: GST-4-Dependent Suppression of Neurodegeneration in C. elegans Models of Parkinson’s and Machado-Joseph Disease by Rapeseed Pomace Extract Supplementation
Source: Front Neurosci. 2019 Oct 17;13:1091. doi: 10.3389/fnins.2019.01091 (PMC6811615; doi:10.3389/fnins.2019.01091)
Supplement: Supplementary file 1 [file Presentation_1.pptx]

## Slide 1
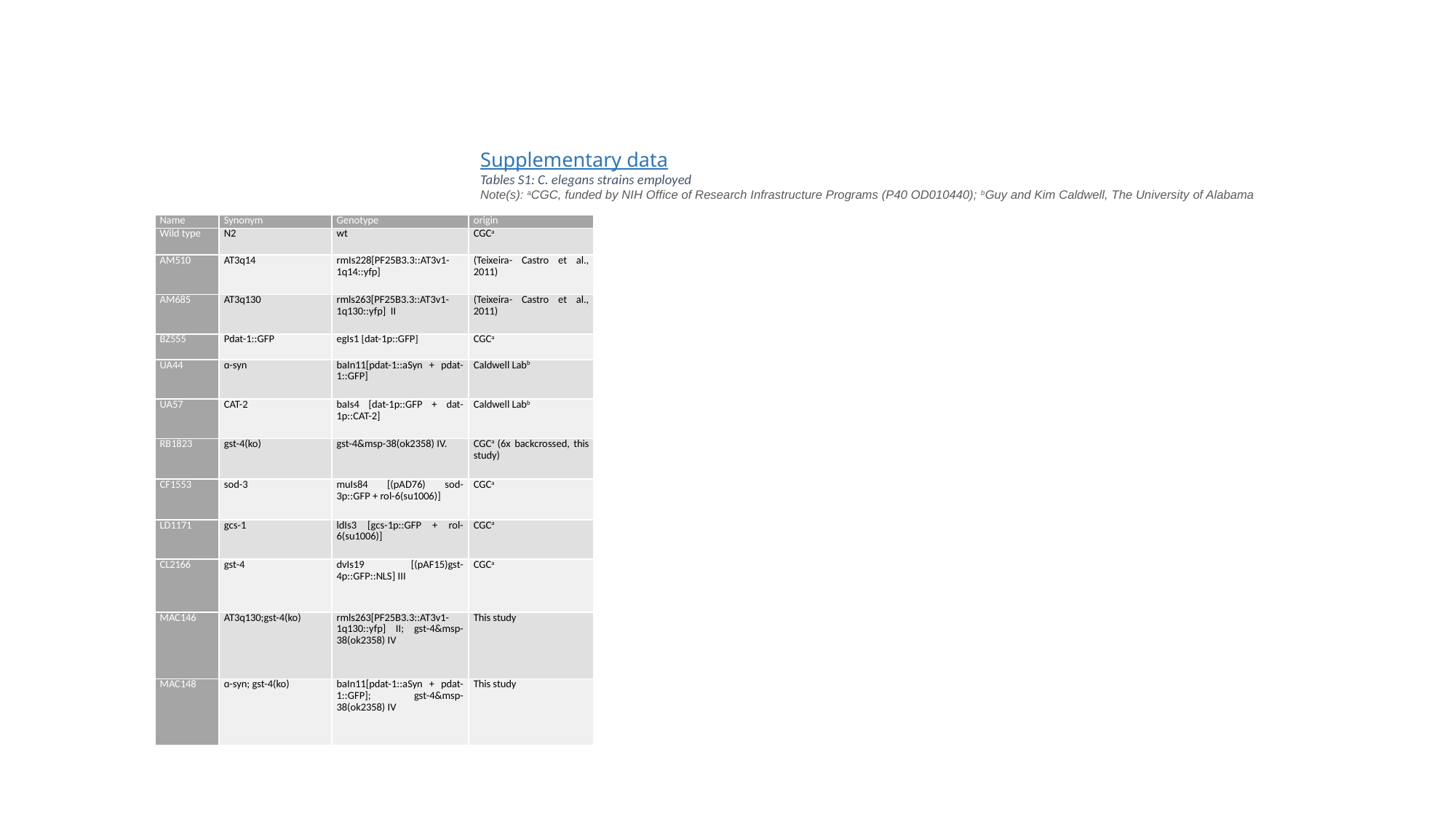

Supplementary data
Tables S1: C. elegans strains employed
Note(s): aCGC, funded by NIH Office of Research Infrastructure Programs (P40 OD010440); bGuy and Kim Caldwell, The University of Alabama
| Name | Synonym | Genotype | origin |
| --- | --- | --- | --- |
| Wild type | N2 | wt | CGCa |
| AM510 | AT3q14 | rmIs228[PF25B3.3::AT3v1-1q14::yfp] | (Teixeira- Castro et al., 2011) |
| AM685 | AT3q130 | rmls263[PF25B3.3::AT3v1-1q130::yfp] II | (Teixeira- Castro et al., 2011) |
| BZ555 | Pdat-1::GFP | egIs1 [dat-1p::GFP] | CGCa |
| UA44 | ɑ-syn | baIn11[pdat-1::aSyn + pdat-1::GFP] | Caldwell Labb |
| UA57 | CAT-2 | baIs4 [dat-1p::GFP + dat-1p::CAT-2] | Caldwell Labb |
| RB1823 | gst-4(ko) | gst-4&msp-38(ok2358) IV. | CGCa (6x backcrossed, this study) |
| CF1553 | sod-3 | muIs84 [(pAD76) sod-3p::GFP + rol-6(su1006)] | CGCa |
| LD1171 | gcs-1 | ldIs3 [gcs-1p::GFP + rol-6(su1006)] | CGCa |
| CL2166 | gst-4 | dvIs19 [(pAF15)gst-4p::GFP::NLS] III | CGCa |
| MAC146 | AT3q130;gst-4(ko) | rmls263[PF25B3.3::AT3v1-1q130::yfp] II; gst-4&msp-38(ok2358) IV | This study |
| MAC148 | ɑ-syn; gst-4(ko) | baIn11[pdat-1::aSyn + pdat-1::GFP]; gst-4&msp-38(ok2358) IV | This study |

## Slide 2
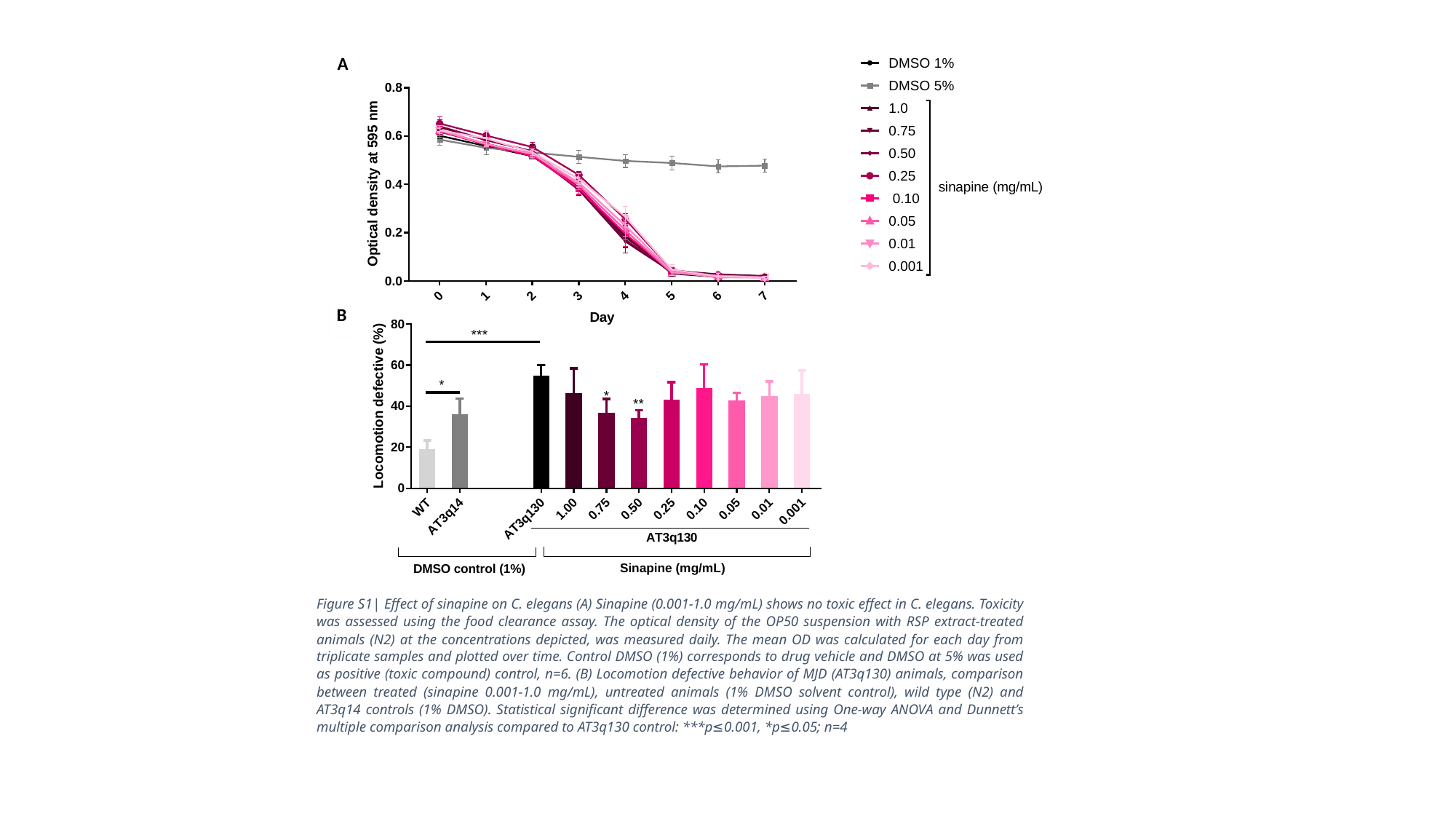

A
B
Figure S1| Effect of sinapine on C. elegans (A) Sinapine (0.001-1.0 mg/mL) shows no toxic effect in C. elegans. Toxicity was assessed using the food clearance assay. The optical density of the OP50 suspension with RSP extract-treated animals (N2) at the concentrations depicted, was measured daily. The mean OD was calculated for each day from triplicate samples and plotted over time. Control DMSO (1%) corresponds to drug vehicle and DMSO at 5% was used as positive (toxic compound) control, n=6. (B) Locomotion defective behavior of MJD (AT3q130) animals, comparison between treated (sinapine 0.001-1.0 mg/mL), untreated animals (1% DMSO solvent control), wild type (N2) and AT3q14 controls (1% DMSO). Statistical significant difference was determined using One-way ANOVA and Dunnett’s multiple comparison analysis compared to AT3q130 control: ***p≤0.001, *p≤0.05; n=4

## Slide 3
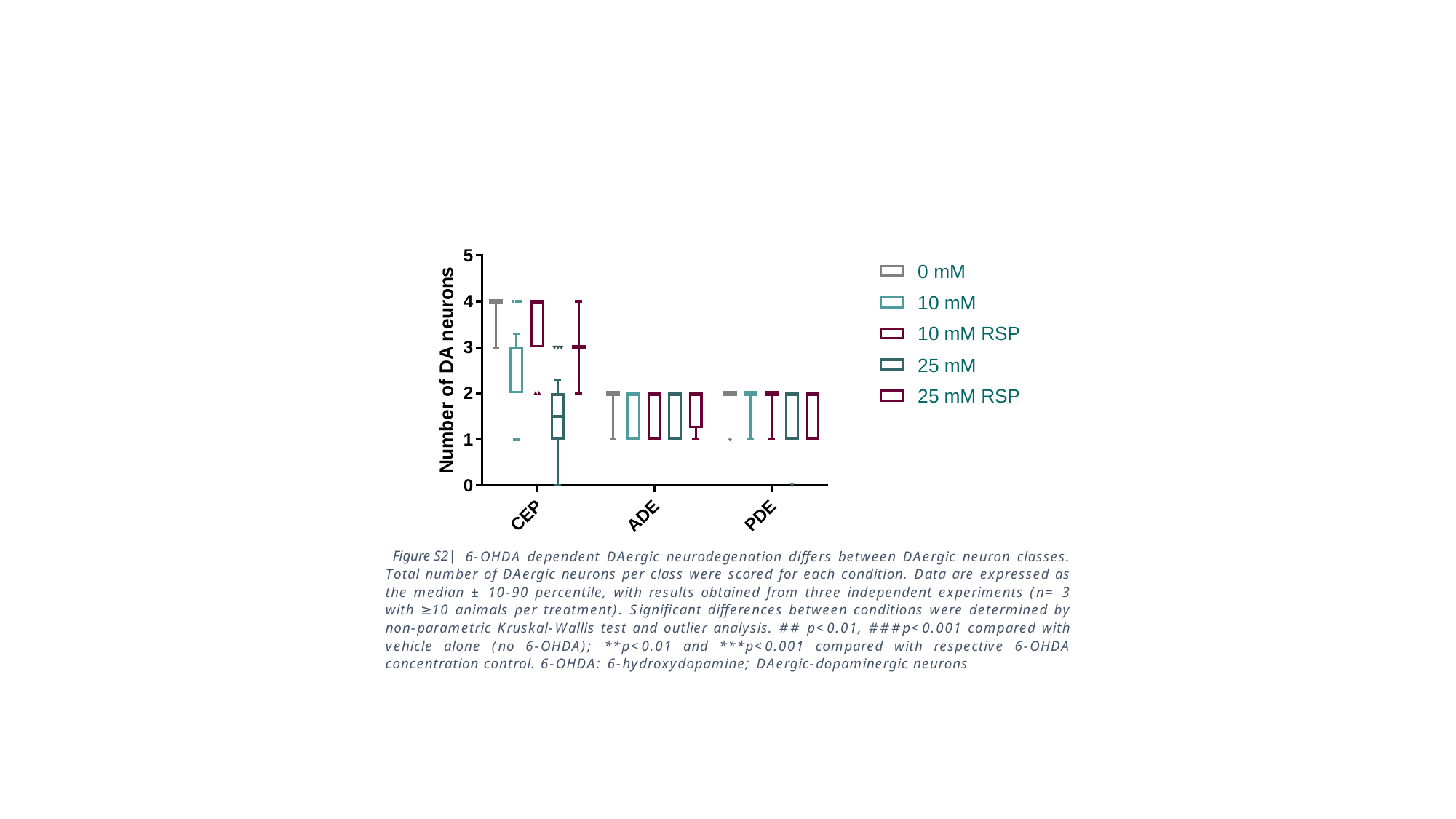

Figure S2|
